# A Simple Method for Enema Administration in One-Day-Old Broiler Chicks

**DOI:** 10.1100/2012/483214

**Published:** 2012-04-30

**Authors:** Guilherme Augusto Marietto-Gonçalves, Fabrizio Grandi, Raphael Lucio Andreatti Filho

**Affiliations:** ^1^Laboratory of Avian Pathology, School of Veterinary Medicine and Animal Science, Universidade Estadual Paulista (UNESP), 18618-970 Botucatu, SP, Brazil; ^2^Department of Pathology, Botucatu Medical School, Universidade Estadual Paulista (UNESP), 18618-970 Botucatu, SP, Brazil; ^3^Laboratory of Investigative and Comparative Pathology, School of Veterinary Medicine and Animal Science, Universidade Estadual Paulista (UNESP), 18618-970 Botucatu, SP, Brazil

## Abstract

The present study aimed to describe a simple technique for enema administration in one-day-old broiler chicks. For this purpose we used 455 unsexed health birds divided into four groups submitted to three different experimental protocols: in the first one, we measured the total length of the large intestine in order to establish a secure distance for probe introduction; in the second, we evaluated maximum compliance of large intestine and diffusion range; finally, based on results obtained we tested the hypothesis in 400 birds in order to standardize the method. Enema solutions applied in an intrarectal manner with a stainless steel gavage BD-10 probe into one-day-old broiler chicks at 0.2 mL at a distance of 1.5 cm proved to be a reliable method.

## 1. Introduction

Enema is a technical procedure that consists in rectal or colonic administration of several drugs that require rapid systemic absorption such as anticonvulsants, analgesics, antiemetics, anesthetic, and antibacterial agents [1]. Plus, it can be used to remove local toxins by flushing or by increasing the volume of liquid which in turn causes rapid expansion of the lower intestinal tract and evacuation [2, 3].

In veterinary medicine, the technique is used both in clinical and experimental settings [4]. In broiler chicks enemas had been used in a diverse set of experiments including toxicological, nutritional, and immunological assays. Despite this, there is no standardized protocol for enema administration in these animals, the majority being empirical.

The objective of this study is to describe a simple method for enema administration that can be applied to one-day-old broiler chickens in order to better standardize further experiments involving this administration route.

## 2. Materials and Methods

### 2.1. Animals

Four hundred fifty-five, one-day-old, unsexed, healthy Cobb Avian Farm broiler chicks (average weight = 42.2 g ± 1.8) were acquired from a commercial incubatory (Céu Azul Alimentos, Pereiras, SP, Brazil) and kept in galvanized wire cages (0, 90 × 0, 60 × 0, 33) in the experimental aviary of the Avian Pathology Laboratory, School of Veterinary Medicine and Animal Science, Universidade Estadual Paulista, UNESP, Botucatu, SP, Brazil. The animals were kept in a controlled environment at a temperature of 27 ± 2°C, a relative humidity of 70 ± 10%, on a 24 h light cycle with a continuous air exchange, and water *ad libitum*. The experiment was approved by the Institutional Ethics Committee of School of Veterinary Medicine and Animal Science, Universidade Estadual Paulista, Botucatu, SP, Brazil.

### 2.2. Animal Experimental Design

The study was divided into three experiments in order to reduce the total number of birds used. The euthanasia procedure used was cervical dislocation, conforming that recommended by Federal Council of Veterinary Medicine, Brazil [5], and Close et al. [6]. All euthanized birds were submitted to necropsy evaluation by a pathologist. Only those birds with no gross signs of disease were considered in the results.

**Experiment 1**

Twenty-five birds were randomly selected and euthanized by cervical displacement technique in order to measure the total length of the large intestine. The last was defined as the distance between cloaca and the ileum-cecum-colon junction at the level of caecal tonsils. Measurement was carried out with an analog caliper (Mitutoyo Sul Americana, Santo Amaro, Brazil). The average length was tabulated and used in the standardization of the second experiment.

**Experiment 2**

Based on the results obtained in the first experiment, thirty animals were randomly selected and divided into two groups (*n* = 15 for each group). Methylene blue dye was administered intrarectally with a stainless steel gavage BD-10 probe (Insight Equipamentos, Ribeirão Preto, SP, Brazil) into broiler chicks at 0.2 or 0.4 mL/animal in order to evaluate the maximum compliance of the large intestine and diffusion range. Signs of dye leakage or rectal evacuation were evaluated up to ten seconds after each enema, after which animals were euthanized and submitted to necropsy.

**Experiment 3**

This final experiment was conducted in accordance with the values obtained in the first two experiments. To this end, four hundred birds were randomly selected and submitted to a saline enema. Signs of solution leakage or rectal evacuation were evaluated up to ten seconds after each enema. Twenty-four hours after instillation, animals were clinically evaluated and euthanized in order to detect gross abnormalities in large intestine.

## 3. Results and Discussion

The average length obtained in Experiment 1 was 1.9 cm (±0.2). Based on this, a standard distance of 1.5 cm was established for the next two experiments.

**Figure 1:**
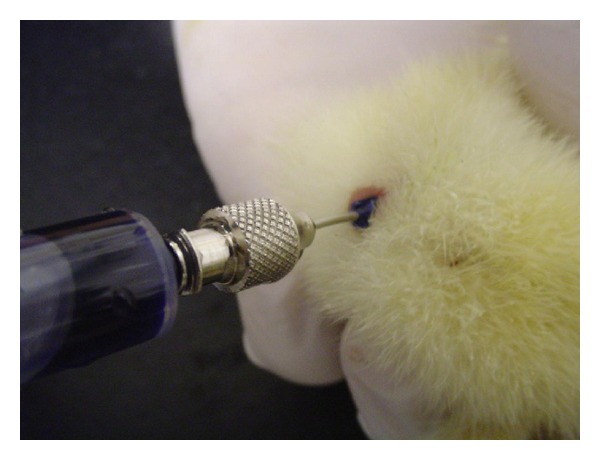
Note leakage of enema in a bird submitted to 0.4 mL methylene blue dye.

**Figure 2:**
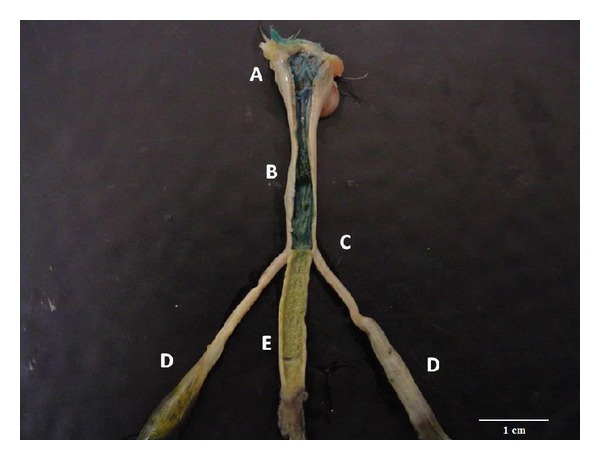
Diffusion range limited to caecal tonsils as evidenced by labeling with the methylene blue dye. A: cloaca; B: rectum; C: caecal tonsils; E: ileum.

In methylene blue dye enema groups (Experiment 2) there were two different results. The group submitted to a 0.2 mL/bird enema did not show signs of leakage or rectal evacuation during or after the procedure. However, those birds submitted to 0.4 mL/bird enema show signs of leakage (Figure 1) and evacuation reflex in 40% and 100%, respectively. All birds (*n* = 30) displayed a diffusion range limited to caecal tonsils as evidenced by labeling with the dye (Figure 2) and no gross abnormalities on necropsy evaluation. The results are summarized on Table 1.

In saline-enema-treated group (Experiment 3), four hundred birds were submitted to 0.2 mL saline enema at an intrarectal distance of 1.5 cm with no signs of leakage and rectal evacuation up to ten seconds after the procedure. There were no clinical signs or gross abnormalities during or after a period of 24 hours, respectively.

Enema administration for routine use in one-day-broiler chicks involves a series of methodological aspects as seen in this study. The total volume of solution administered should not exceed the maximum capacity retention (compliance) of the lower segments of large intestine, that is, rectum and colon, and therefore was determined in 0.2 mL/bird. The administration of dye at 0.4 mL/bird induces retrograde cloacal reflux and rectal evacuation reflex in some animals which can compromise the total absorbed drug dose in a clinical setting or experimental assay. Additionally, the interval between defecation and enema administration plays a central role in the maintenance of drugs in lower intestinal portions and consequently their absorption [4].

The distance for the introduction of the probe was inferior (1.5 cm) compared to the average distance between cloaca and ileum-cecum-colon junction (1.8 cm ±0.2). The former was adopted as the standard distance for Experiments 2 and 3 in order to avoid anatomical limits and therefore lesions to intestinal tract as could be confirmed by necropsy which reveal no gross lesions in all birds evaluated (*n* = 430).

Clinical applicability of the technique described here includes the administration of drugs and experimental inoculums (e.g., biological agents), radiological contrasts for large intestine analysis, collection of fecal samples for microbiological, immunological (e.g., IgA), and/or parasitological assays.

In conclusion, enema solutions administered with a stainless steel gavage BD-10 probe into one-day-old broiler chicks at 0.2 mL/bird at an intrarectal distance of 1.5 cm proved to be a reliable method. Technical data generated by this study can be applied safely for experimental assays involving young broiler chicks.

## Figures and Tables

**Table 1:** Total number of birds showing cloacal reflux, rectal evacuation, or gross abnormalities following methylene blue dye instillation.

Sign	Volumes	
	0.2 mL	0.4 mL
Cloacal reflux	0% [0/15]*	40% [6/15]
Rectal evacuation	0% [0/15]	100% [15/15]
Gross abnormalities	0% [0/15]	0% [0/15]

*Positive number of animals/total number of birds.

## References

[1] Van Hoogdalem EJ, De Boer AG, Breimer DD (1991). Pharmacokinetics of rectal drug administration—part I. General considerations and clinical applications of centrally acting drugs. *Clinical Pharmacokinetics*.

[2] Nelson RW, Couto CG (2010). *Medzicina Interna de Pequenos Animais*.

[3] Sakate M, Nogueira RMB, Andrade SF, Andrade SF (2008). Terapêutica das intoxicações. *Manual de Terapêutica Veterinária*.

[4] Smith T, Smith A, Kelly J (1998). The practical aspects of repeated administration of an enema to beagle dogs. *Toxicology Letters*.

[5] Brasil http://www.furb.br/site/arquivos/357080-486559/Resolucao%20714_%202002.htm.

[6] Close B, Banister K, Baumans V (1996). Recommendations for euthanasia of experimental animals—part 1. *Laboratory Animals*.

